# Interventional Management of Acute Pancreatitis and Its Complications

**DOI:** 10.3390/jcm14186683

**Published:** 2025-09-22

**Authors:** Muaaz Masood, Amar Vedamurthy, Rajesh Krishnamoorthi, Shayan Irani, Mehran Fotoohi, Richard Kozarek

**Affiliations:** 1 Division of Gastroenterology and Hepatology, Center for Digestive Health, Virginia Mason Franciscan Health, Seattle, WA 98101, USA; 2 Department of Interventional Radiology, Virginia Mason Franciscan Health, Seattle, WA 98101, USA; 3 Center for Interventional Immunology, Benaroya Research Institute, Virginia Mason Franciscan Health, Seattle, WA 98101, USA

**Keywords:** endoscopy, interventional radiology, acute pancreatitis, pancreatic necrosis, pseudocyst, percutaneous drainage, endoscopic ultrasound, pancreatic duct leak, pseudoaneurysm, embolization

## Abstract

Acute pancreatitis (AP) is the most common cause of gastrointestinal-related hospitalizations in the United States, with gallstone disease and alcohol as the leading etiologies. Management is determined by disease severity, classified as interstitial edematous pancreatitis or necrotizing pancreatitis, with severity further stratified based on local complications and systemic organ dysfunction. Regardless of etiology, initial treatment involves aggressive intravenous fluid resuscitation with Lactated Ringer’s solution, pain and nausea control, early oral feeding in 24 to 48 h, and etiology-directed interventions when indicated. In gallstone pancreatitis, early endoscopic retrograde cholangiopancreatography (ERCP) with sphincterotomy is indicated in the presence of concomitant cholangitis or persistent biliary obstruction, with subsequent laparoscopic cholecystectomy as standard of care for stone clearance. The role of interventional therapy in uncomplicated AP is limited in the acute phase, except for biliary decompression or enteral feeding support with nasojejunal tube placement. However, in severe AP with complications, interventional radiology (IR) and endoscopic approaches play a pivotal role. IR facilitates early percutaneous drainage of symptomatic, acute fluid collections and infected necrosis, particularly in non-endoscopically accessible retroperitoneal or dependent collections, improving outcomes with a step-up approach. IR-guided angiographic embolization is the preferred modality for hemorrhagic complications, including pseudoaneurysms. In the delayed phase, walled-off necrosis (WON) and pancreatic pseudocysts are managed with endoscopic ultrasound (EUS)-guided drainage, with direct endoscopic necrosectomy (DEN) reserved for infected necrosis. Dual-modality drainage (DMD), combining percutaneous and endoscopic drainage, is increasingly utilized in extensive or complex collections, reflecting a collaborative effort between gastroenterology and interventional radiology comparable to that which exists between IR and surgery in institutions that perform video assisted retroperitoneal debridement (VARD). Peripancreatic fluid collections may fistulize into adjacent structures, including the stomach, small intestine, or colon, requiring transpapillary stenting with or without additional closure of the gut leak with over-the-scope clips (OTSC) or suturing devices. Additionally, endoscopic management of pancreatic duct disruptions with transpapillary or transmural stenting plays a key role in cases of disconnected pancreatic duct syndrome (DPDS). Comparative outcomes across interventional techniques—including retroperitoneal, laparoscopic, open surgery, and endoscopic drainage—highlight a shift toward minimally invasive approaches, with decreased morbidity and reduced hospital stay. The integration of endoscopic and interventional radiology-guided techniques has transformed the management of AP complications and multidisciplinary collaboration is essential for optimal patient outcomes.

## 1. Introduction

The incidence of acute pancreatitis is rising in North America and Europe. There are several factors which may contribute to the increased incidence of AP including obesity rates, alcohol consumption, improved diagnostic modalities, and regional variations. AP is more common in older adults. The diagnosis of acute pancreatitis (AP) requires 2 of 3 following criteria: (1) abdominal pain, (2) serum amylase or lipase >3 times the upper limit of normal, or (3) characteristic findings on abdominal imaging [1]. The most common causes of AP include gallstones in 40% to 70% of cases and alcohol which accounts for approximately one-third of cases. Gallstone pancreatitis is an acute event with or without antecedent biliary colic. A diagnosis can usually be made with ultrasonography. Alcohol-associated AP should be on the differential diagnosis in patients with several years of moderate-to-heavy alcohol use [2]. Pancreatitis is not due to the amount and type of alcohol consumed, and only about 5–10% of patients with alcohol use disorder develop it. Environmental factors such as diet, smoking, and genetic predisposition play a role as cofactors [3]. About 5% of cases occur due to primary and secondary hypertriglyceridemia, although this figure is higher in patients with pre-existing and poorly controlled diabetes mellitus. A serum triglyceride level should be checked in the absence of gallstones or significant history of alcohol use and may be the etiology if the triglyceride level is greater than 1000 mg/dL. Pancreatic cancer should be excluded in patients older than 40 who present with idiopathic pancreatitis [4]. In some patients, an etiology may not be known, and further workup and follow-up imaging should be pursued.

Up to one-third of patients with AP have moderate-to-severe disease. Moderate severity is defined as transient organ failure, which resolves within 48 h and/or development of complications such as peripancreatic fluid collections. Severe acute pancreatitis is defined as the presence of organ failure lasting over 48 h. Risk factors for severe AP include baseline elevated BUN and hematocrit as well as comorbidities, obesity, and the presence of systemic inflammatory response syndrome (SIRS). Anatomically, AP can be classified as interstitial, usually mild, or necrotizing pancreatitis with moderate to severe disease. Interstitial pancreatitis presents as focal or diffuse enlargement of the pancreas with enhancement on contrast CT scans. Inflammatory fat stranding can be noted in peripancreatic tissue. Necrotizing pancreatitis involves diffuse or focal areas of non-viable pancreatic parenchyma with or without involvement of peripancreatic fat necrosis. The necrosis is initially sterile and can eventually become infected, often within one to two weeks [5,6]. Mortality doubles if there is organ failure in addition to infected pancreatic necrosis (IPN). We outline the evolving interventional, non-surgical management of acute pancreatitis and its complications in this narrative review.

## 2. Materials and Methods

### 2.1. Endoscopic Retrograde Cholangiopancreatigraphy in Acute Pancreatitis

**Figure 1:**
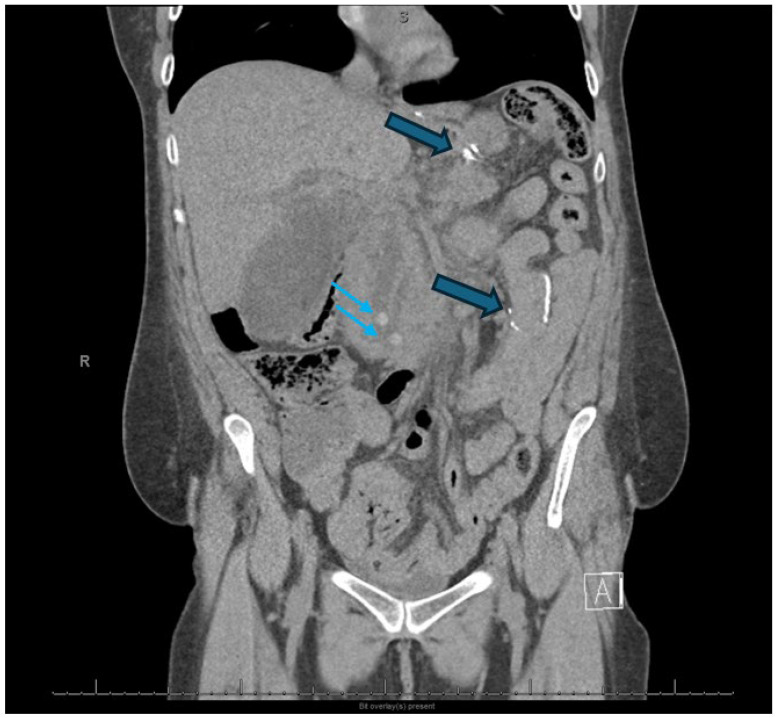
Small arrows demonstrate retained common bile duct stones in this patient with a Roux-en-Y gastric bypass, intact gallbladder, and gallstone pancreatitis. Larger arrows outline gastric staple line and roux limb, respectively. Post-operative anatomy makes endoscopic retrograde cholangiopancreatography access difficult requiring balloon-assisted enteroscopy or an endoscopic ultrasound-directed transgastric endoscopic retrograde cholangiopancreatography (EDGE) procedure demonstrated in Scheme 1.

**Scheme 1:**
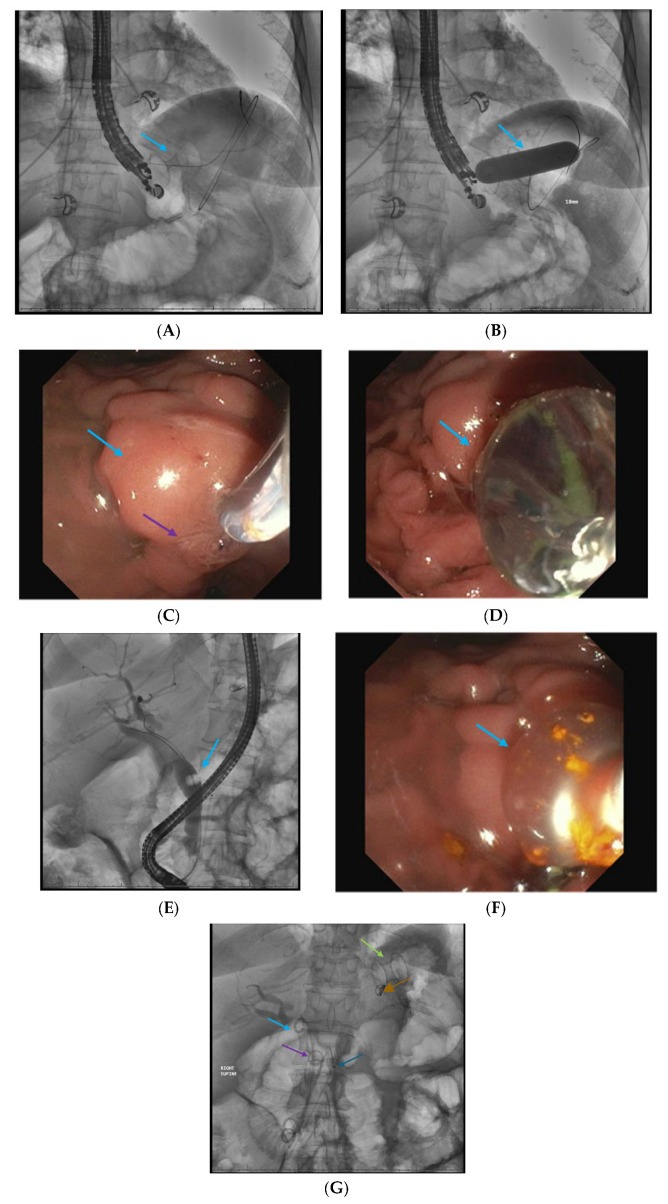
Endoscopic ultrasound-directed transgastric endoscopic retrograde cholangiopancreatography (EDGE) procedure in the patient depicted in Figure 1—A gastrogastrostomy was performed to the excluded stomach using a 20 mm lumen apposing metal stent (LAMS) (arrow) (**A**), followed by balloon dilation of the stent to 18 mm to allow endoscopic access to the duodenum (**B**). Of note, there was an impacted stone (blue arrow) in the papilla which was treated with a small sphincterotomy (purple arrow) (**C**) and a balloon sphincteroplasty (**D**,**E**) (blue arrows). Following balloon retrieval of the stones (**F**), 2 biliary stents were placed. Final radiograph (**G**) demonstrated indwelling biliary stents (blue and purple arrows), PD stent (teal arrow) placed to minimize the risk of post-ERCP pancreatitis, and the GG LAMS (green arrow) which had been secured in place by an Ovesco clip (Ovesco Endoscopy AG, Tübingen, Germany) (brown arrow) to minimize the risk of LAMS dislodgement during a complex procedure.

The majority of patients with biliary pancreatitis have passed their stones by the time they seek medical care. In patients with gallstone pancreatitis and an elevation in bilirubin, ERCP is potentially indicated if there is documentation of a persistent CBD stone, contingent on institutional expertise. An imaging study to include MRCP or EUS is necessary to confirm retained intraductal stones, and ERCP alone should not be used as a diagnostic procedure [Table 1]. Previous studies suggest that patients with severe pancreatitis and those with ascending cholangitis are most likely to benefit from early ERCP and biliary sphincterotomy to decompress the biliary tree [7] [Figure 1; Scheme 1]. In a nationwide study of 70,030 patients, inpatient ERCP for acute biliary pancreatitis without cholangitis was associated with a lower all-cause mortality. ERCP within 24 h and 72 h was associated with improved clinical outcomes and lower healthcare costs [8]. However, the American College of Gastroenterology guidelines recommend medical therapy over ERCP in the first 72 h in acute biliary pancreatitis without cholangitis [9]. Patients should undergo cholecystectomy to reduce the risk of recurrent AP.

Finally, it is common practice in the community setting to perform ERCP in patients with retained common bile duct stones prior to laparoscopic cholecystectomy, even in patients with mild pancreatitis. This is usually performed during the index hospitalization in conjunction with laparoscopic cholecystectomy and may allow for stabilization of an acutely or chronically ill patient and surgery when the patient has been stabilized. In institutions with expertise in laparoscopic transcystic common bile duct exploration (LCBDE), the trend has been to approach retained stones laparoscopically in medically fit patients at the time of gallbladder removal. Studies demonstrate the efficacy of transcystic LCBDE with success rates for duct clearance comparable to ERCP, decreased rates of post-procedural pancreatitis, and shorter duration of hospitalization [10,11,12,13].

### 2.2. Interventional Radiology-Guided Percutaneous Drainage of Early Pancreatic Fluid Collections

Although many patients with acute pancreatitis develop peripancreatic fluid collections (PFC), most cases spontaneously resolve and do not require interventional therapy. In patients with persistent fever, multiorgan dysfunction, increased WBC or CRP levels, and a contrast CT that fails to demonstrate other possible sources of infection, early percutaneous drainage (PCD) of a PFC should be considered [Table 2]. CT can define the number and locations of multiple PFCs and the presence or absence of a wall surrounding the collection, as well as allow estimation of the amount of necrotic material, graded as <30%, 30–50% and >50%. A CT severity index (CTSI) was first described by Balthazar et al. in 1994 to distinguish mild, moderate, and severe acute pancreatitis on a scale from 0 to 10 [14]. However, the original Balthazar scoring system had several limitations including no strict correlation with complications, i.e., organ failure, and variable application and interpretation among users [15]. The modified CTSI was published in 2004 and has been demonstrated to have a stronger correlation with patient outcomes [16].

Although some necrotic collections tend to encapsulate earlier than 4 weeks and are amenable to endoscopic drainage, PCD can be performed earlier with the assistance of CT using the Seldinger technique. Access is confirmed by contrast injection, and a hydrophilic guidewire is placed in each of the necrotic collections through the most direct transperitoneal route, avoiding bowel and solid organs. A dilating catheter is advanced over the wire, and the tract is dilated. Subsequently, a double sump 12–16 Fr catheter is placed into the cavity, and fluid is aspirated (Scheme 1).

After successful placement, the catheter is flushed with saline to irrigate the cavity. In some instances, continuous irrigation using a liter of normal saline is performed. Depending on the clinical course, the percutaneous catheter can be subsequently upsized after 24 to 72 h to a maximum of 28 to 30 Fr double-sump large-bore catheters with distal holes over time [17]. Abundant irrigation is performed according to the size of the PFC. Catheters are changed periodically or if drainage decreases. Injection of contrast into the cavity can also aid in the diagnosis of intestinal fistulae. CT scans are performed after 1–2 weeks to check the efficacy of the drainage [18]. PCD is not typically performed for acute peripancreatic fluid collections unless there is a significant likelihood of infection as they often undergo spontaneous resolution. MRI and US are more sensitive for solid material in a collection compared to CT scans.

### 2.3. Infected Pancreatic Necrosis

**Scheme 2:**
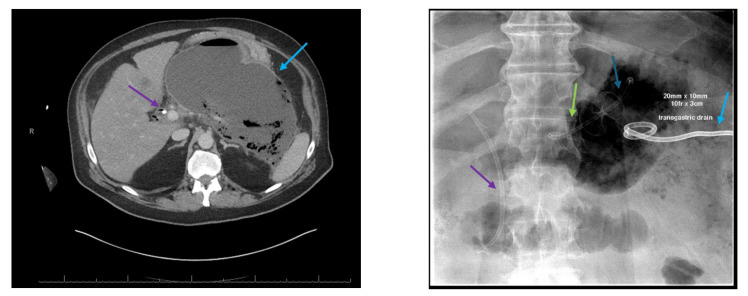
(**Left**) Axial view of computed tomography scan of the abdomen with contrast demonstrated large area of walled-off pancreatic necrosis (blue arrow) in a patient who remained febrile despite intravenous antibiotics. Note previously placed biliary stent (purple arrow) placed for biliary decompression. (**Right**) Fluoroscopic image with a percutaneous transgastric drain (blue arrow) into the walled-off pancreatic necrosis. Note previous biliary stent (purple arrow) placed for biliary decompression and a 20 mm × 10 mm lumen-apposing metal stent (dark blue arrow) with a 10 Fr × 3 cm pigtail stent (green arrow) placed immediately after percutaneous transgastric drainage (dual modality drainage).

**Figure 2:**
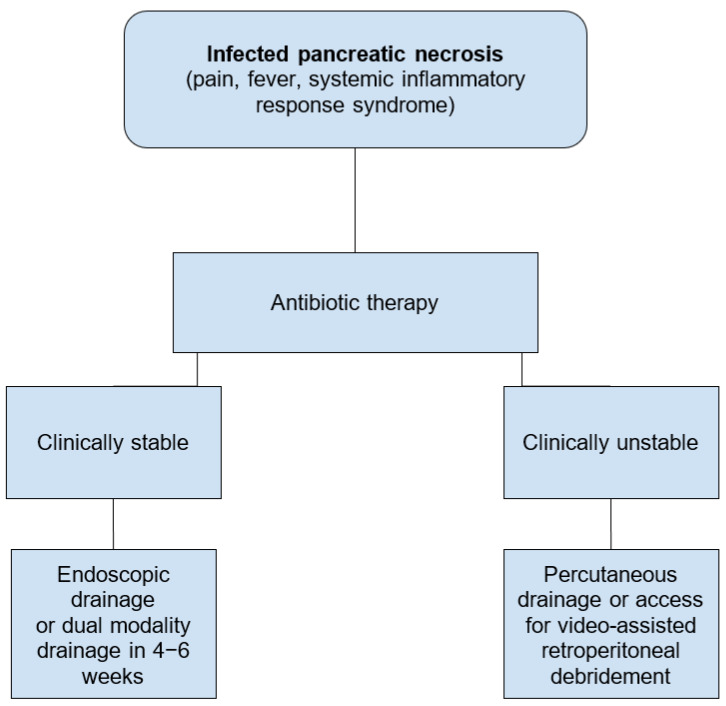
Current Guidelines for Infected Pancreatic Necrosis.

The management of IPN has been a long-standing topic of debate. Historically, IPN was treated with open surgical debridement, which was associated with significant patient mortality ranging from as low as 14% in individuals in whom treatment was delayed 4–6 weeks after presentation to 69% for those operated on acutely [19]. If there is concern for infected pancreatic necrosis, initiation of broad-spectrum antibiotics has been recommended to include carbapenems, quinolones, metronidazole, and third- or fourth-generation cephalosporins [20]. Necrotizing pancreatitis often requires multidisciplinary management which involves advanced endoscopists, interventional radiologists, and surgeons, and a step-up approach that may include percutaneous drainage, endoscopic drainage, or surgical debridement [Table 2 and Table 3; Figure 2; Scheme 2] [21]. In clinically stable patients, delaying surgical drainage to over four weeks has been associated with decreased mortality [22,23]. The American College of Gastroenterology suggests against fine needle aspiration of IPN [9].

In the PANTER trial, the step-up approach was used as a bridge to surgery. The trial involved percutaneous drainage (PCD) followed by video-assisted retroperitoneal debridement (VARD). More than a third of patients with IPN did not require surgery and were managed with PCD alone. A step-up approach with the intention to avoid surgery led to a success rate of 69% [24]. One limitation of the study is that it did not provide a direct comparison between VARD and open necrosectomy in cases of failed PCD. Samanta et al., in turn, demonstrated in a study of 218 patients that EUS-guided drainage of WON, infected or otherwise, resulted in higher clinical success (92.1% vs. 64.6%, *p* < 0.0001), as well as higher (*p* = 0.007) and faster (*p* < 0.0001) resolution of organ failure compared to PCD. Additionally, it was recommended that PCD should be avoided in WON with >40% solid components [25].

In an additional retrospective study of 47 patients comparing percutaneous drainage, endoscopic drainage, internal derivation, and necrosectomy in the late phase of moderate to severe AP, all of these modalities were demonstrated to be effective options based on the patient’s clinical status and the location of the necrosis. However, an up-front invasive approach was associated with a worse quality of life [26]. Ebrahim et al. documented outcomes in 144 patients with WON > 15 cm. Most patients (93%) were treated with endoscopic transluminal drainage, and only 1 patient (0.7%) required open necrosectomy. Procedural adverse events occurred in 7% of patients, and 24% of patients died during admission from multiorgan failure. The study concluded that minimally invasive treatment of large WON is feasible with a minimal need for surgery and acceptable rates of morbidity and mortality [27]. Baroud et al. explored the role of a protocolized endoscopic drainage with a lumen apposing stent (LAMS) compared to a control group treated according to clinical preference. The protocol required repeat cross-sectional imaging within 14 days after LAMS placement and regular endoscopic necrosectomies if WON diameter reduction was <50%. While the number of necrosectomies was similar, the protocol group was associated with faster WON resolution, lower AEs, shorter ICU stay, and fewer days of nutritional support [28]. Many of those patients who develop necrosis have been shown to have long-term problems including new-onset diabetes or exocrine pancreatic insufficiency, as well as recurrent attacks of pancreatitis. In a study of 373 patients with necrotizing pancreas who had a median follow-up of 13.5 years, 26% were readmitted for recurrent pancreatitis. Endocrine insufficiency, which occurred in 36% of patients, and exocrine insufficiency, which occurred in 38% of patients, were both less frequent with conservative treatment (*p* < 0.001 and *p* = 0.016, respectively). Pancreatic necrosis of >50% was demonstrated to be an important predictor of interventions and complications during follow-up [29].

In a randomized controlled trial of 183 patients, upfront necrosectomy at the index intervention instead of as a step-up intervention was associated with a significantly reduced number of reinterventions in patients with infected necrotizing pancreatitis and fully encapsulated collections [30]. However, the number of study participants was small. The study had an open-label nature which can result in bias. Additionally, 21% of patients in the step-up group had treatment success without necrosectomy which may challenge the appropriateness of upfront necrosectomy for all patients [31].

Finally, Avudiappan et al. compared minimal incision retroperitoneal necrosectomy (MIRN), conventional open necrosectomy, and VARD. MIRN was associated with low postoperative mortality and low postoperative stay, and there was no significant difference in re-intervention rate, postoperative bleeding, and enterocutaneous fistula between the 3 groups [32].

### 2.4. Endoscopic Ultrasound-Guided Techniques for Walled-Off Pancreatic Necrosis (WON)

The first step in the management of infected necrosis is antibiotic therapy. It is recommended to delay invasive interventions for at least 4 weeks after the initial presentation to allow for liquefaction and encapsulation of the necrotic collection. A well-encapsulated cavity allows for safe drainage with reduced risk of pneumoperitoneum or pneumoretroperitoneum. However, in some patients, the necrotic collections can wall off completely in less than 4 weeks of the initial episode. Endoscopic drainage of collections in early stages is reserved for patients with significant clinical deterioration. Although endoscopic drainage is technically feasible, it is challenging due to the presence of solid necrotic content as opposed to liquified content in a walled-off cavity which often occurs in the delayed phase [33]. If early drainage is warranted, patients should be referred to a tertiary care center with expertise in interventional radiology, therapeutic endoscopy, critical care, and surgery to manage pancreatic complications. EUS provides detailed imaging of a pseudocyst or a necrotic cavity and its contents. Intervening vessels should be avoided during the access procedure. Once a safe site has been located, the cyst is punctured using an electrocautery-assisted lumen apposing metal stent. Once the stent has been deployed, a guidewire is advanced into the cavity and coiled. Two double-pigtail stents are placed coaxially into the LAMS to keep food debris out of the cavity as well as prevent stent migration into or out of the WON cavity. The cystgastrostomy or cystduodenostomy leads to continuous internal drainage and results in the collapse of the cavity and closure of the pancreatic fistula. A CT scan is usually performed at 2–4 weeks to evaluate for resolution of the cavity and fistula. If there is no resolution, the LAMS is removed, and the two double-pigtail stents are left to maintain the tract. In cases where patients have a significant PD stricture precluding passage of a wire, EUS-guided pancreaticogastrostomy or pancreaticoduodenostomy may be considered. During this procedure, a puncture of the PD is performed, thereby creating a transgastric fistula, and a guidewire is placed into the main PD. Following this, ductal decompression is achieved by the placement of transgastric stents [34]. These latter techniques are reserved for individuals with a dilated pancreatic duct and those who present with a flare of acute superimposed on chronic pancreatitis.

### 2.5. Direct Endoscopic Necrosectomy

**Scheme 3:**
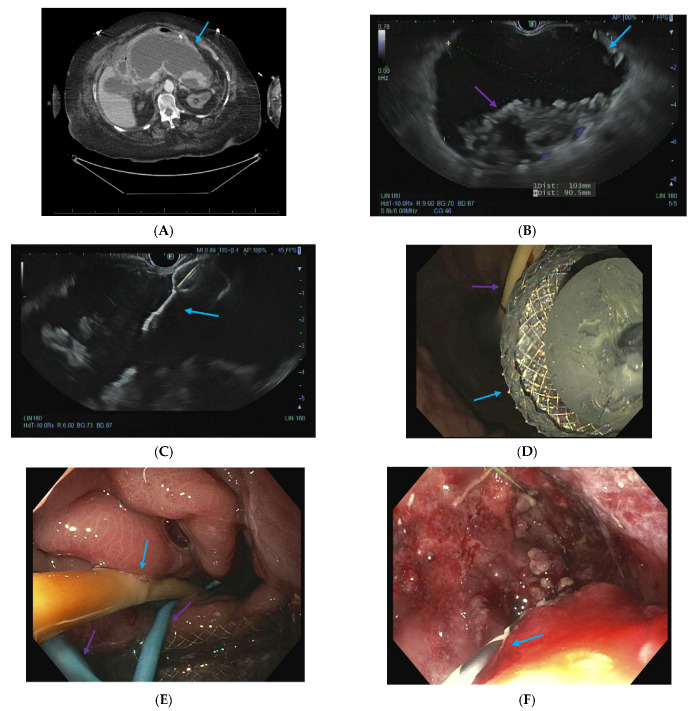

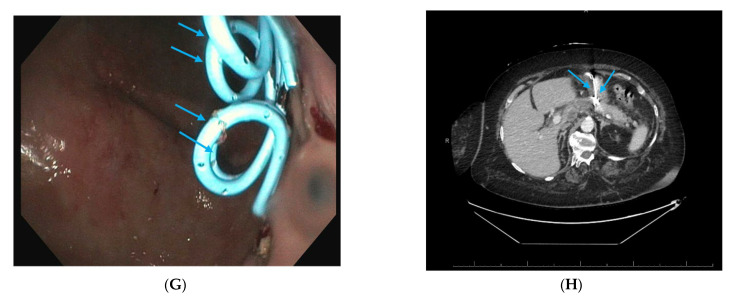
(**A**) Axial computed tomography scan of the abdomen with contrast revealed a large area of walled-off pancreatic necrosis (arrow) in a patient with biliary pancreatitis and gallstones. (**B**) Endoscopic ultrasound demonstrated a large fluid collection (blue arrow) with necrotic debris (purple arrow). (**C**) Arrow demonstrated delivery of a lumen-apposing metal stent (LAMS) (arrow) into the necrotic cavity. (**D**) Endoscopic image of LAMS (blue arrow). Of note, there was a nasojejunal feeding tube in addition to the LAMS (purple arrow). (**E**) Following LAMS removal and placement of pigtail stents (purple arrows), a direct endoscopic necrosectomy was performed with large jaw forceps (blue arrow). (**F**) Endoscopic view of the base of the necrotic cavity over a guidewire (blue arrow). (**G**) Four double pigtail stents were placed in the necrotic cavity (blue arrows). (**H**) Axial computed tomography scan of the abdomen with contrast which revealed a collapsed area of walled-off pancreatic necrosis with double pigtail stents (arrows) in place.

Necrosectomy is currently performed endoscopically using a combination of mechanical debridement using snares, forceps, baskets, or nets. It is a labor-intensive process, and a lack of specialized equipment adds to the challenge of whether performed per os or by VARD. DEN usually requires multiple sessions requiring passage through a metal stent or a fistulous tract that may be at a challenging angle and increase the risk of stent dislodgement (Scheme 3). Novel solutions include use of cap-assisted necrosectomy, Ovesco AG, a large over-the-scope grasper, and EndoRotor (Interscope, Inc., Northbridge, MA, USA), a device that can fulgurate the necrotic material into smaller pieces.

EndoRotor is a three-in-one tool that allows simultaneous dissection, resection, and extraction of necrotic material. It consists of a console unit that delivers cutting, suction, and irrigation with a footswitch control through a 3.1 mm diameter flexible catheter. The catheter has an open window at the distal tip and an inner cannula that can rotate at 1000 or 1750 revolutions per minute. The system uses a high-performance vacuum pump to help with aspiration. The inner cannula of the EndoRotor catheter has teeth that rotate and debride necrotic tissue, which is then aspirated and collected in a vacuum canister. The case series by Rizzatti et al. demonstrated the use of EndoRotor for DEN in four patients who previously had incomplete attempts at DEN [35].

Extra-large graspers such as the Ovesco excavator (OTSG Xcavator^TM^—Ovesco Endoscopy AG, Tübingen, Germany), are additional new tools that are attached to the tip of the endoscope for necrosectomy. The device is made of transparent plastic with a diameter of 14.7 mm when closed, allowing entry into large caliber LAMS. In some cases, once the fistula matures, it can be dilated, and the scope can be advanced into the cavity after removing the LAMS. Although the grasping tool is easy to open in tight spaces or open partially, it is not possible to navigate into branched foxhole-like cavities, due to the rigid nature of the device [36].

### 2.6. Dual Modality Drainage

Alternatively, necrotic cavities with debris can be treated with both endoscopic stent placement and a simultaneous JP drain placed percutaneously to allow irrigation and facilitate internal drainage. Percutaneous drains can also be placed into non-contiguous or distant anatomic sites, such as the pelvic gutters, or into sites that are not accessible endoscopically (refer to Scheme 2).

In our institution, we have frequently used a hybrid technique that includes both an endoscopic and percutaneous drainage, particularly in patients with multiple areas of WON or in patients who have a component of necrosis not accessible endoscopically. In a study of 278 patients comparing early and late dual modality drainage (DMD) for necrotic fluid collections (NFCs), the technical success, clinical success, procedural, and early post-procedural adverse events were similar [37]. There were 2 deaths in the early drainage and 9 deaths in the late drainage group, *p* = 0.991. In a systematic review and meta-analysis of 9 studies and 855 patients, the complication rates and the number of patients requiring subsequent necrosectomies were similar in the early drainage and late drainage groups, although mortality was slightly higher in the early drainage group. The study concluded that early drainage can be performed if needed in selected patients, and a multidisciplinary team is needed if early drainage is utilized [38].

### 2.7. Interventional Radiology-Guided Embolization of Pseudoaneurysms

**Figure 3:**
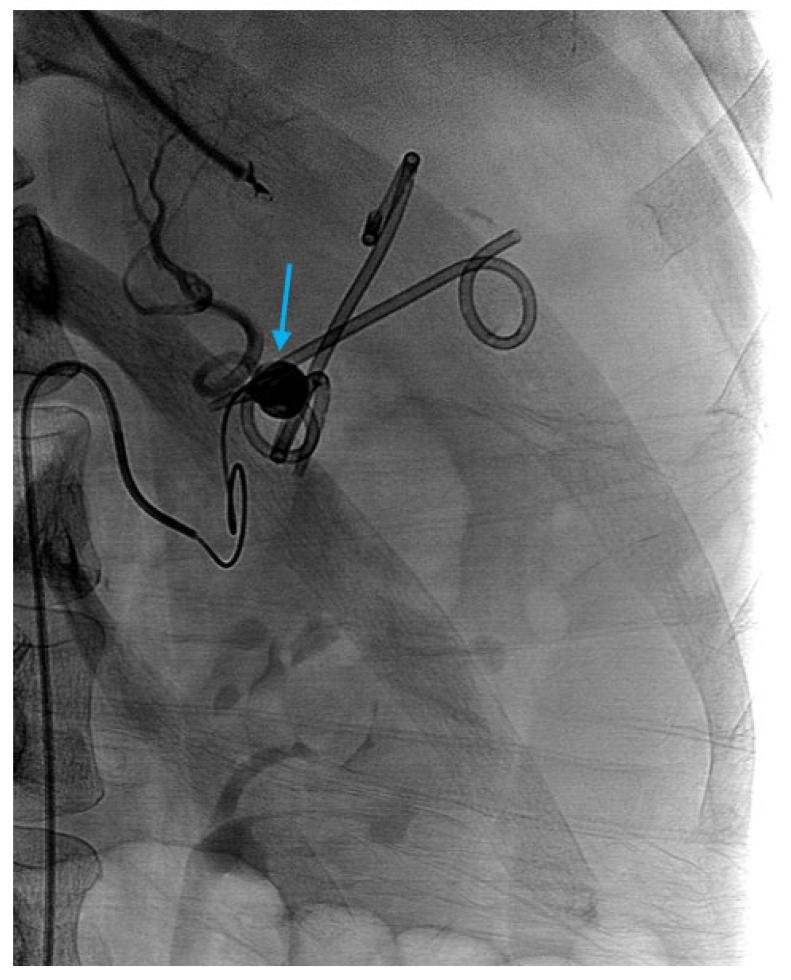
Fluoroscopic image with arrow which demonstrates embolization of a splenic artery aneurysm and its feeding vessels. The standard of care involves embolization of the parent artery with coil or gel foam. Note pigtail stents at a previous lumen apposing metal stent (LAMS) drainage site. Prolonged LAMS retention has been associated with an increased risk of erosion into contiguous blood vessels.

LAMS are preferred in the management of peripancreatic fluid collections as the wider diameter allows for rapid drainage and collapse of the necrotic cavity. However, LAMS may lead to formation of pseudoaneurysm due to the abrasion of the distal flange associated with acute inflammation [Figure 3]. Bleeding from pseudoaneurysms from LAMS erosion may occur in the first 2 weeks and require an early removal [39]. Pseudoaneurysms, however, commonly occur de novo in severe disease, contiguous to a JP drain or in the setting of necrosis of the pancreatic tail, and involve the splenic artery. In a study of 3048 patients with AP, 808 patients (26.5%) had vascular complications which included visceral vein thrombosis, portal hypertension, and arterial complications, and 95 patients (11.8%) had bleeding. Several independent risk factors were noted such as male gender, hyperlipidemia, recurrent disease, tobacco use, WBC count, additional non-vascular local complications, CTSI, and APACHE II score [40]. Madhusudhan et al. reported a study of 141 patients which explored outcomes of radiological interventions of hemorrhagic complications in patients with acute and chronic pancreatitis. The difference in the technical success rates (98.2%, 95% CI 94.6–101.8% vs. 96.7%, 95% CI 92.1–101.3%), clinical success rates (84%, 95% CI, 73.5–94.5% vs. 83.9%, 95% CI 74–93.9%) and complications of embolization (8%; 95% CI, 0.2–15.8% vs. 19.6%; 95% CI, 8.9–30.4%) were not statistically significant between the acute and chronic pancreatitis groups. Recurrence and long-term outcomes were also similar between the two groups. However, patients with AP had a significantly higher mortality of 50%, due to sepsis and organ failure, compared to patients with chronic pancreatitis, who had a mortality of 1.8% (*p* < 0.001) [41].

Pseudoaneurysms are contained in the adventitia of the artery and are associated with a high risk of mortality. Patients may present with a history of pancreatitis and abdominal pain, drop in hemoglobin, or gastrointestinal bleeding. Although diagnostic work-up includes endoscopy to rule out mucosal bleeding in the gastrointestinal tract amenable to endoscopic therapy, the absence of a potential bleeding site should raise concern for hemorrhagic pancreatitis or a pseudoaneurysm and prompt an urgent computed tomography angiography. Interventional radiology-guided embolization of pseudoaneurysms is the mainstay of treatment in hemodynamically stable patients. Early angiography with localization and embolization of pseudoaneurysms with embolic coils and gel foam is associated with improvement in survival, and a decrease in mortality from 90% to 15–50% [Figure 3] [42,43]. Surgery is reserved for hemodynamically unstable patients or patients who have failed embolization.

### 2.8. Pancreatic Fistula Treatment

**Scheme 4:**
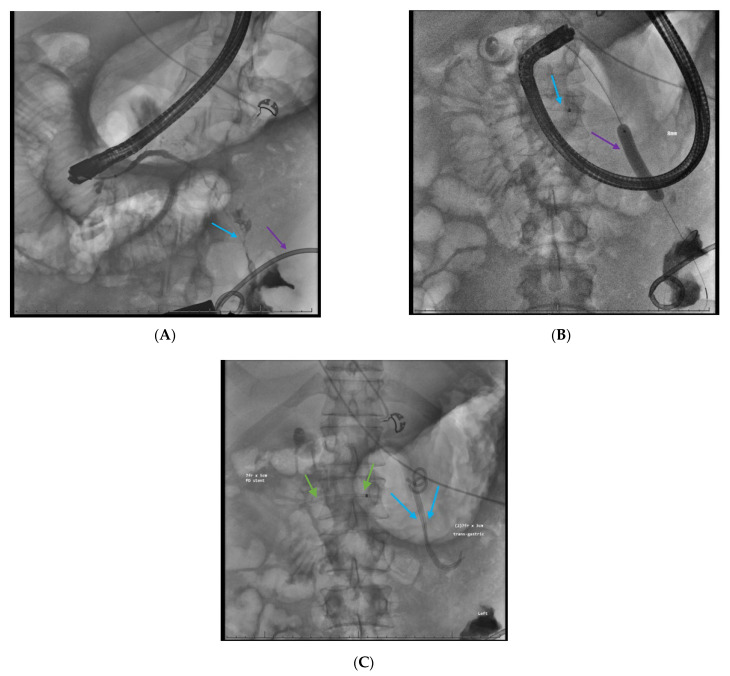
(**A**) Fluoroscopic image demonstrated a pancreatic duct leak (**A**) (blue arrow) from the tail of the pancreas into the left pelvic gutter and a percutaneous drain in place (purple arrow). Following placement of a 7 Fr × 5 cm pancreatic duct stent (**B**) (blue arrow) and (**C**) (green arrows) and endoscopic ultrasound-guided transgastric access into the necrotic fluid collection, the tract was balloon dilated (purple arrow) followed by placement of 7 Fr × 5 cm pigtail stents (blue arrows) (**C**) to allow for drainage. This is a variation of dual modality drainage in the setting of an ongoing disruption of the duct in the pancreatic tail.

Disruption of the pancreatic duct can range in severity from involvement of a ductal side branch to partial or complete disruption of the main pancreatic duct [Scheme 4]. Pancreatic secretions leak and form peripancreatic fluid collections, such as acute PFCs, and when they mature, a pseudocyst is formed. Pancreatic secretions can digest peripancreatic tissues and form connections into the peritoneum, small bowel, colon or stomach, and rarely into pleural or pericardial spaces. The fistulous tract can cause amylase- and protein-rich fluid collections which result in pancreatic ascites or pleural fluid with elevated amylase. These are considered internal fistula formations [44]. In a systematic review and meta-analysis of 6 studies and 157 patients with pancreaticopleural fistulas, the success rate for endoscopic treatment was 79.2% and the recurrence rate was 1.88%. Two mortalities were reported in the study including one post-surgical death. The study recommended medical and endoscopic therapy initially, especially in specialized centers, and prompt surgical intervention if medical or endoscopic therapy fails [45].

Extravasated pancreatic secretions can also leak to the skin surface, such as at the site of surgical incision following debridement or the PCD site which forms a pancreaticocutaneous fistula. Pancreaticocutaneous fistulas occur more commonly when only a single modality of external drainage is performed. The anatomy of the pancreatic duct may impact the approach to management. Ductal disruptions can be aggravated and perpetuated by downstream obstruction of the PD [46]. Partial duct disruptions can be treated by endoscopic therapy such as pancreatic sphincterotomy and stent placement [Scheme 4]. Pancreatic sphincterotomy increases the size of the PD orifice, thereby removing the point of resistance and improving transpapillary flow of secretions.

### 2.9. Approach to Disconnected Pancreatic Duct Syndrome (DPDS) After Severe Pancreatitis

DPDS is defined as complete disruption of the PD with an upstream viable pancreas. The excluded tail portion of the pancreas continues to cause leakage of pancreatic secretions which results in recurrent fluid collections or pancreatitis in the orphaned portion of the gland. A delay in diagnosis of DPDS has been associated with increased morbidity, duration of hospitalization, and costs [47]. DPDS has historically required a distal pancreatectomy which may be difficult in the setting of splenic vein thrombosis and left-sided portal hypertension. Extravasation of contrast from the PD on ERCP confirms the diagnosis of DPDS, although it also can be diagnosed with a secretin MRCP [48]. Long-term placement of double pigtail stents (DPS) allows patency of the cystgastrostomy tract. In a retrospective study, placement of long-term, transmural double pigtail stents after PFC resolution was an effective modality to prevent recurrence of PFCs and may be beneficial in patients with DPDS where the excluded pancreas drains exclusively through the transgastric fistula kept patent by the pigtail stents [49]. In a meta-analysis of 30 studies, endoscopic transmural drainage was superior to transpapillary drainage for the management of DPDS [50].

### 2.10. Acute on Chronic Pancreatitis—Fibrotic PD Strictures or Calculi

While inflammatory PD strictures and duct disruptions are associated with acute pancreatitis, fibrotic PD strictures or calculi typically occur in chronic pancreatitis [51,52,53]. Fibrotic PD strictures or calculi in the proximal duct can force the secretions to the site of leakage rather than at the papilla. Endoscopic therapies such as dilation and stenting can assist in forward flow. Dilation balloons with a diameter of 4–6 mm are used to dilate strictures over a guidewire. When strictures cannot be traversed with balloon dilation, graduated dilation catheters, which range from 3 to 10 Fr in size, can be advanced over the guidewire. The placement of a pancreatic stent serves several purposes. First, a pancreatic stent is placed to maintain patency of strictures which were dilated. Secondly, the proximal end of the stent can be positioned upstream to the site of disruption, allowing closure of the ductal defect by diverting flow. Lastly, pancreatic stents bridge the pancreatic orifice and reduce resistance encountered at the Sphincter of Oddi. Pancreatic stent diameters range from 5 Fr to 10 Fr, which is determined based on the size of the PD. In cases where the stricture cannot be traversed, a shorter stent is placed. Longer stents are selected, such as one with the external flap outside the PD orifice and the internal flap above the site of ductal disruption.

### 2.11. Outcomes in Quality of Life and Cost Effectiveness

Acute necrotizing pancreatitis is associated with a substantial disease burden. In a study of 373 patients with necrotizing pancreatitis, the quality of life scores did not differ between conservative, endoscopic or percutaneous drainage alone, and the necrosectomy groups [29]. In a single-center randomized controlled trial of 66 patients, the endoscopic transluminal approach compared with minimally invasive surgery was associated with significantly reduced mean total costs and increased physical health scores for quality of life [54]. Tran et al. reported that open debridement compared to endoscopic or percutaneous drainage only and endoscopic or laparoscopic debridement was associated with increased in-hospital complications, duration of hospitalization, and costs [55]. Frailty in acute pancreatitis, assessed using the Frailty Risk Score, has been associated with increased complications, higher mortality, and increased healthcare costs [56].

## 3. Conclusions

While various interventional techniques have been outlined, it is important to note that institutional expertise and availability of resources may impact the choice of intervention. Some endoscopists may not have expertise with EUS, and some surgeons may not have experience with LCBDE. Moreover, there may be challenges with reimbursement for EUS-guided procedures or combined cholecystectomy and LCBDE. Patients with complications of acute pancreatitis should be referred to high-volume centers in which IR, advanced therapeutic endoscopy, and surgery teams are experienced in the multidisciplinary approach and have the required resources to address severe acute pancreatitis and its complications [57].

The majority of patients with acute pancreatitis require support as opposed to interventional therapy. Patients with uncomplicated gallstone pancreatitis should undergo laparoscopic cholecystectomy, ideally during their index hospitalization. In those with retained CBD stones, stone removal should be contingent upon the expertise and skillset of both the surgeon and the therapeutic endoscopy team. Patients with severe disease should be assessed by a team that includes gastroenterologists/therapeutic endoscopists, surgeons, interventional radiologists, and hospitalists or intensivists. Those with acute cholangitis should undergo early biliary decompression, most commonly with ERCP. In those who develop necrosis, current studies demonstrate that they should be managed conservatively for 4–6 weeks before undergoing drainage. However, in the setting of infected necrosis unresponsive to antibiotics or clinical deterioration, percutaneous drainage, at times in conjunction with VARD, is indicated. Moreover, additional interventions to include embolization of a bleeding pseudoaneurysm or decompressive laparotomy for abdominal compartment syndrome may be required. Although it may be dependent on the institution, the treatment of pancreatic pseudocysts and WON has evolved from open or laparoscopic surgery to EUS-guided drainage with LAMS. Direct endoscopic necrosectomy through the LAMS tract as well as dual modality drainage for irrigation are performed for the resolution of the collections. DMD also plays a key role in draining anatomic areas not amenable to endoscopic access. ERCP, in turn, is performed to treat biliary obstruction from distal CBD compression as well as for the placement of bridging PD stents in patients with a PD leak. Regardless of the treatment modality, patients who develop necrosis or have severe disease are at increased risk for recurrent pancreatitis as well as pancreatic exocrine and endocrine insufficiency.

## Figures and Tables

**Table 1:** The Role of Endoscopic Retrograde Cholangiopancreatography in Acute Pancreatitis.

Biliary pancreatitis	Concomitant cholangitis Persistent choledocholithiasis Post-cholecystectomy Pre-laparoscopic cholecystectomy
Severe acute pancreatitis	Biliary stent for common bile obstruction Pancreatic duct stent for pancreatic leak
Biliary pancreatitis with cholecystitis and a poor surgical candidate	Transpapillary gallbladder stent placement

**Table 2:** The role of interventional radiology in acute pancreatitis.

Acute pancreatic fluid collections	Drain placement
Enteral nutrition	Placement of a feeding tube
Acute symptomatic pancreatic fluid collections	Percutaneous drainage
Necrotic fluid collections	Dual modality drainage Percutaneous access for video-assisted retroperitoneal debridement
Pseudoaneurysm	Embolization using coils and gel foam
Obstructive jaundice and a failed endoscopic retrograde cholangiopancreatography	Percutaneous transhepatic biliary drainage versus endoscopic ultrasound access
Pancreatitis with acute cholecystitis and a poor surgical candidate	Percutaneous cholecystostomy Percutaneous transhepatic biliary drainage versus endoscopic ultrasound access

**Table 3:** The role of endoscopic ultrasound in acute pancreatitis.

Diagnosis	Neoplasm Retained stone Infected fluid collections
Walled-off necrosis	EUS-guided drainage Dual modality drainage
Pancreatic pseudocyst	EUS-guided drainage
Disconnected pancreatic duct syndrome	Endoscopic ultrasound +/− endoscopic retrograde cholangiopancreatography and interventional radiology
Pancreatitis with acute cholecystitis and a poor surgical candidate	EUS-guided cholecystoduodenostomy
Gastric outlet obstruction	EUS-guided gastroenterostomy
Altered anatomy	EDGE (EUS-directed transgastric endoscopic retrograde cholangiopancreatography)

## Data Availability

Data has been included in the article.
